# Acquired hemophilia A in the setting of dual anticoagulation therapy and lupus anticoagulant: a case report

**DOI:** 10.1186/s13256-022-03402-x

**Published:** 2022-05-03

**Authors:** Victor Chen, Lauren C. Roby, Stephanie Wentzel, Mingjia Li, Nicholas Jones

**Affiliations:** 1 The Ohio State University College of Medicine, Columbus, OH USA; 2 The Ohio State University Hematology-Oncology Fellowship, Columbus, OH USA; 3 Division of Hospital Medicine, Department of Internal Medicine, The Ohio State University, M112 Starling Loving Hall, 320 W. 10th Avenue, Columbus, OH 43210 USA

**Keywords:** Hemophilia A, Factor VIII deficiency (acquired), Lupus anticoagulant, Chromogenic Bethesda assay, Case report

## Abstract

**Background:**

Acquired hemophilia A is a disorder caused by autoantibodies against coagulation factor VIII that may present with severe bleeding. We report a rare case of acquired hemophilia A presenting with coexisting lupus anticoagulant.

**Case presentation:**

An 81-year-old Caucasian female presented with large ecchymoses over the torso and extremities in the setting of an enoxaparin bridge to warfarin. Anticoagulation was held, but she continued to develop bruises with significant anemia and prolonged coagulation studies that failed to correct with mixing. Workup revealed factor VIII activity < 1% and a positive lupus anticoagulant. Initial testing for a factor VIII inhibitor was confounded by the presence of lupus anticoagulant, requiring a chromogenic Bethesda assay to confirm the presence of the inhibitor, establishing the diagnosis of acquired hemophilia A. The patient was initially treated with oral prednisone 80 mg daily and factor VIII inhibitor bypassing activity 25 units/kg twice daily before transitioning to susoctocog alfa 50 units/kg twice daily after placement of a tunneled line for outpatient rituximab infusions. On discharge, the patient’s ecchymoses were resolving and factor VIII levels improved. Following completion of rituximab therapy, the patient’s factor VIII activity normalized and factor VIII inhibitor was suppressed.

**Conclusions:**

Diagnosis of acquired hemophilia A can be confounded by other causes of abnormal coagulation studies and may require specialized testing, such as a chromogenic Bethesda assay, to confirm the presence of a factor VIII inhibitor.

## Background

Acquired hemophilia A is a rare disorder caused by the development of autoantibodies against coagulation factor VIII. Timely recognition and diagnosis are important as the condition may present with life-threatening bleeding. We report a case of acquired hemophilia A presenting with severe ecchymoses in the setting of dual anticoagulation therapy and presence of lupus anticoagulant (LA).

## Case presentation

**Fig. 1.**
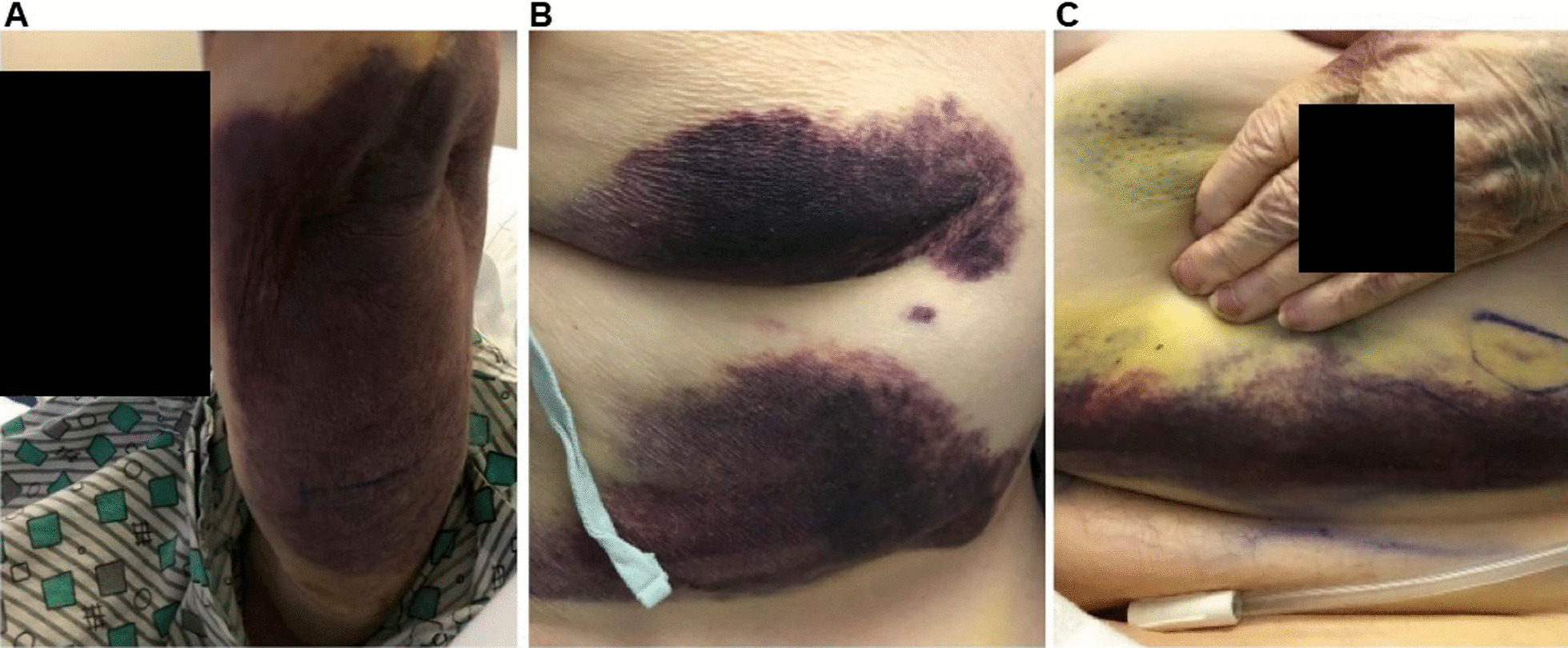
Extensive ecchymoses due to acquired hemophilia A. **A** Ecchymosis of left arm. **B** Ecchymosis of left flank. **C** Ecchymosis of lower abdomen, below panniculus

An 81-year-old Caucasian female, with a history significant for atrial fibrillation complicated by recurrent stroke on warfarin with the most recent stroke over 5 years ago, well-controlled rheumatoid arthritis, and hypertension, presented with multiple large ecchymoses over the torso and extremities in the setting of an enoxaparin bridge to warfarin for a subtherapeutic international normalized ratio (INR) (Fig. 1). On admission, workup was notable for hemoglobin of 5.7 g/dL, prothrombin time (PT) 28.1 seconds (s), INR 2.7, partial thromboplastin time (PTT) > 180 seconds, D-dimer of 1.02 μg/mL (normal < 0.5 μg/mL), and normal fibrinogen. Anticoagulation was held, but she continued to develop bruises daily with persistently elevated PTT > 180 seconds that failed to correct with mixing studies, raising concern for a factor deficiency. Further workup revealed factor VIII activity < 1% (normal 75–220%). Given her prolonged PTT, LA testing was performed and found to be positive. Initial testing for a factor VIII inhibitor using the standard Bethesda assay was confounded by the presence of LA, requiring a specialized chromogenic Bethesda assay, which confirmed the presence of the inhibitor at 61 Bethesda units (normal ≤ 0.5 units). The patient was diagnosed with acquired hemophilia A on the basis of the presence of a factor VIII inhibitor. She did not meet diagnostic criteria for antiphospholipid syndrome (APS) despite testing positive for LA; additional testing was negative for anticardiolipin and β_2_-glycoprotein antibodies. Given the association between acquired factor VIII inhibitors and malignancy, additional screening for concomitant malignancy was performed. Computed tomography of the chest, abdomen, and pelvis was unremarkable. Serum protein electrophoresis suggested an inflammatory picture but was otherwise unremarkable. A bone marrow biopsy performed during a future unrelated hospital admission was also unremarkable.

The patient was started on oral prednisone 80 mg daily with addition of factor VIII inhibitor bypassing activity (FEIBA) 25 units/kg twice daily. FEIBA was transitioned to susoctocog alfa 50 units/kg twice daily after placement of a tunneled line for outpatient administration of rituximab. With initiation of treatment, the patient’s ecchymoses began to resolve, and no new bruising was noted. Following discharge, the patient received four cycles of weekly rituximab and completed a simultaneous steroid taper. At completion of therapy, her factor VIII levels normalized and factor VIII inhibitor was suppressed (Table 1). The patient continues to follow with hematology with no evidence of relapse and has not yet restarted anticoagulation therapy given this recent diagnosis.

**Table 1:** Laboratory findings

Test	At presentation	After anticoagulation washout	After steroids	After rituximab	Reference
PT (seconds)	28.1	20.0	12.5	14.2	11.9–14.2
INR	2.7	1.7	1.0	1.1	0.9–1.1
PTT (seconds)	> 180.0	127.6	69.4	33.2	24.0–34.3
Factor VIII activity (%)	< 1	–	117	247	75-220
Factor VIII inhibitor (Bethesda units)	–	61	–	0.9	≤ 0.5

*PT* prothrombin time, *INR* international normalized ratio, *PTT* partial thromboplastin time

## Discussion

Acquired hemophilia A is a rare disorder caused by development of autoantibodies to coagulation factor VIII. The annual incidence of acquired hemophilia A is approximately one case per million people [1]. This condition most commonly presents in the elderly and may be associated with pregnancy, autoimmune conditions, malignancy, and certain medications; however, many cases lack a clear underlying cause [2]. Acquired hemophilia A can present acutely with severe soft tissue bleeding but generally does not cause the hemarthroses classically associated with congenital hemophilia A [2]. Initial workup will reveal prolonged PTT that fails to correct with mixing studies and a decreased factor VIII level. Diagnosis is confirmed by detection of a factor VIII inhibitor, typically via the standard Bethesda assay in which patient plasma is incubated with normal plasma and residual factor VIII activity is measured via a clotting assay [3].

In this patient, diagnosis was confounded by several factors that also cause abnormal coagulation. Her bleeding was initially attributed to recent dual anticoagulation therapy with enoxaparin and warfarin. In these cases, mixing studies with either protamine sulfate correction or following washout of anticoagulation, which may require up to 2 days, are needed to exclude the effect of heparin on prolonged PTT. Persistently prolonged PTT should prompt testing for alternative etiologies such as the presence of a factor VIII inhibitor or LA. Coexisting factor VIII inhibitor and LA is a rare phenomenon that has been reported to manifest with bleeding such as nontraumatic hematomas, even in the presence of underlying APS [4–9]. LA prolongs PTT and mimics factor VIII inhibitors in standard clotting assays; the presence of both antibodies will result in impaired detection of the factor VIII inhibitor. Alternatives to the standard Bethesda assay have been developed, including chromogenic assays (as used in this patient), enzyme-linked immunosorbent assays, and fluorescence immunoassays [10]. The chromogenic Bethesda assay measures residual factor VIII activity via a chromogenic substrate and is unaffected by the presence of heparin, direct thrombin inhibitors, and LA [11]. As such, it is a more favorable assay for detection of a factor VIII inhibitor when other agents that prolong PTT are present and was required for diagnosis in this patient with coexisting factor VIII inhibitor and LA.

Treatment of acquired hemophilia A falls into three main categories: increasing factor VIII levels, factor VIII bypassing agents, and inhibitor suppression [2]. Factor VIII levels may be increased by desmopressin or factor VIII replacement; however, these treatments are typically used only in patients with very low titer inhibitors [2]. Factor VIII bypassing agents are used for patients with higher titer inhibitors and include porcine recombinant factor VIII and FEIBA [2]. Lastly, the inhibitor may be targeted via immunosuppressive therapies such as glucocorticoids, cyclophosphamide, cyclosporine, and rituximab [2]. Roughly 70–90% of patients achieve at least partial remission with treatment by 1 year, with higher initial factor VIII levels associated with better prognosis [12]. Remission may also occur spontaneously, and inhibitors may persist or relapse after treatment cessation.

Resuming anticoagulation therapy following a bleed requires an assessment of bleeding and clotting risks. For nonhemophilic patients on anticoagulation who develop a gastrointestinal or intracranial bleed, current recommendations support reinitiating anticoagulation therapy [13]. There is less evidence for patients with acquired hemophilia, although some guidelines suggest that, following normalization of factor levels, antiplatelet or anticoagulation therapy should be restarted [14].

## Conclusion

Acquired hemophilia A is a rare disorder that can present with bleeding and prolonged PTT. Diagnosis may be confounded by anticoagulation therapy and the presence of LA. Bleeding out of proportion to anticoagulation should raise suspicion for a factor deficiency and prompt factor level measurements. Patients should be referred to a hematologist with expertise in bleeding and thrombotic disorders for management, particularly for risk–benefit analysis regarding reinitiation of anticoagulation therapy.

## Data Availability

Not applicable.

## Abbreviations

LA: Lupus anticoagulant
INR: International normalized ratio
PT: Prothrombin time
PTT: Partial thromboplastin time
APS: Antiphospholipid syndrome
FEIBA: Factor VIII inhibitor bypassing activity
